# Parasite-Related Genetic and Epigenetic Aspects and Host Factors Influencing *Plasmodium falciparum* Invasion of Erythrocytes

**DOI:** 10.3389/fcimb.2018.00454

**Published:** 2019-01-14

**Authors:** Monica Ararat-Sarria, Manuel A. Patarroyo, Hernando Curtidor

**Affiliations:** ^1^Receptor-Ligand Department, Fundación Instituto de Inmunología de Colombia, Bogotá, Colombia; ^2^PhD Programme in Biomedical and Biological Sciences, Universidad del Rosario, Bogotá, Colombia; ^3^Molecular Biology and Immunology Department, Fundación Instituto de Immunología de Colombia (FIDIC), Bogotá, Colombia; ^4^School of Medicine and Health Sciences, Universidad del Rosario, Bogotá, Colombia

**Keywords:** malaria, *Plasmodium falciparum*, invasion factors, regulatory mechanism, phenotype change

## Abstract

Malaria, a disease caused by *Plasmodium* parasites, is widespread throughout tropical and sub-tropical regions worldwide; it mostly affects children and pregnant woman. Eradication has stalled despite effective prevention measures and medication being available for this disease; this has mainly been due to the parasite's resistance to medical treatment and the mosquito vector's resistance to insecticides. Tackling such resistance involves using renewed approaches and techniques for accruing a deep understanding of the parasite's biology, and developing new drugs and vaccines. Studying the parasite's invasion of erythrocytes should shed light on its ability to switch between invasion phenotypes related to the expression of gene sets encoding proteins acting as ligands during target cell invasion, thereby conferring mechanisms for evading a particular host's immune response and adapting to changes in target cell surface receptors. This review considers some factors influencing the expression of such phenotypes, such as *Plasmodium*'s genetic, transcriptional and epigenetic characteristics, and explores some host-related aspects which could affect parasite phenotypes, aiming at integrating knowledge regarding this topic and the possible relationship between the parasite's biology and host factors playing a role in erythrocyte invasion.

## Introduction

Human malaria occurs after the bite of a female *Anopheles* mosquito carrying *Plasmodium* parasites; the disease emerges mostly in tropical and sub-tropical regions around the world. Five *Plasmodium* species infect humans: *P. falciparum, P. vivax, P. ovale, P. malariae*, and *P. knowlesi* (*P. falciparum* being the most pathogenic and most frequently associated with mortality) (WHO, 2017). The World Health Organization (WHO) has reported a slight increase in the amount of cases of the disease worldwide, rising from 211 million in 2015 to 216 million in 2016, despite an overall tendency to become decreased having been observed during the last few years (WHO, 2017). Such increase has been linked to an expansion of *Plasmodium*'s resistance to drugs, thereby urging the development of more effective treatment and prevention strategies, such as vaccines (WAMIN Consortium Authors et al., 2016; Amato et al., 2018). Understanding the parasite's biology and its relationship with a host's interaction will help in depicting its behavior during invasion and indicate effective methods for developing improved ways of avoiding its development in a particular host (Weiss et al., 2015; Patarroyo et al., 2016).

Most of malaria's clinical symptoms are related to its ability to select, invade, and proliferate inside erythrocytes. A pool of proteins acting as invasion ligands having affinity for receptors on erythrocyte surface would thus enable the recognition, attachment and invasion of the parasite in its merozoite form (Weiss et al., 2015). The parasite can enable the differential expression of its invasion ligands by recognizing host cell surface receptors; these have redundancy in their function, leading to the emergence of different invasion pathways denominated invasion phenotypes (Gaur et al., 2004; Stubbs et al., 2005).

Many specific invasion phenotypes have been described for *Plasmodium falciparum* strains, mostly being related to the invasion-associated protein families of erythrocyte binding antigens (*Pf* EBAs, associated with *pfeba175, pfeba140, pfeba181*, and *pfebl1* genes) and reticulocyte binding-like homologs (*Pf* Rhs, related to *pfrh1, pfrh2a, pfrh2b, pfrh4*, and *pfrh5* genes) (Iyer et al., 2007; Tham et al., 2012). These phenotypes have also been classically correlated with sialic acid-dependent or -independent invasion patterns (Dolan et al., 1990). Sialic acid-dependent parasites are essentially associated with greater expression of ligands needing sialic acid moieties in erythrocyte membrane receptors, such as members of the *Pf* EBA protein family (i.e., *Pf* EBA175, *Pf* EBA140, *Pf* EBA181, *Pf* EBL1) and some members of the *Pf* Rh family (i.e., *Pf* Rh1) (Cowman et al., 2017). Parasite ligands which do not need these moieties for binding to erythrocyte surface receptors prevail in sialic-acid-independent parasites (i.e., some members of the *Pf* Rh protein family, like *Pf* Rh2b and *Pf* Rh4) (Dolan et al., 1990; Nery et al., 2006; Ochola-Oyier et al., 2016; Cowman et al., 2017). It is also known that the parasite can switch from one invasion phenotype to another (depending on a particular host's environment or culture conditions) by varying the expression of its key invasion ligands (Stubbs et al., 2005; Awandare et al., 2018).

Studies attempting to explain the switch mechanism involved in the parasite invasion phenotype have suggested that invasion phenotypes could result from mutations in invasion-related genes or fluctuations in such genes' transcription (Duraisingh et al., 2003). Some studies have also shown that modifications in the parasite's environment can induce changes in invasion gene epigenetic regulation; nevertheless, the specific mechanisms affecting such genes' transcription and epigenetic regulation is still not well understood (Bowyer et al., 2015). Moreover, selective pressure by a host's immune system and variability regarding host cell surface receptor expression could be associated with the emergence of these phenotypes (Abdi et al., 2016, 2017). This review has considered the available knowledge regarding some parasites' genetic aspects influencing the development of invasion phenotypes, such as transcriptional and epigenetic characteristics, looking for a better understanding of the factors involved in erythrocyte invasion leading to such diversity, taking into account the effect of some host-related factors, such as host immune response and erythrocyte surface receptors.

## Malaria and Parasite Invasion

A deeper knowledge of this parasite's biology is necessary considering that *P. falciparum* infection is associated with higher than average morbidity and mortality regarding the remaining species (as it could invade all erythrocyte stages and produce high parasitaemia, inducing severe anemia, acidaemia and cerebral malaria) (Imtiaz et al., 2015); its study is fundamental in elucidating the key steps of its lifecycle inside a human host (WAMIN Consortium Authors et al., 2016). The parasite's invasion of erythrocytes marks the erythrocytic phase of infection which begins with the parasite sensing and first attaching itself to a host erythrocyte. This is followed by reorientation and erythrocyte invasion by parasite invagination followed by formation of the parasitophorous vacuole (PV) (Cowman et al., 2017). The parasite will transform into a ring form inside the PV, then become a trophozoite followed by a schizont form which will burst and release a fresh load of merozoite which will invade other erythrocytes, thereby maintaining the erythrocytic cycle of infection which is directly associated with malaria's clinical symptoms (Oakley et al., 2011; WAMIN Consortium Authors et al., 2016; Mangal et al., 2017). Establishing a continuous *in vitro Plasmodium falciparum* parasite culture during erythrocytic phase has facilitated the study of merozoite interactions with erythrocytes (Trager and Jensen, 1976; Thompson et al., 2001; Radfar et al., 2009).

Continuous culture-related results from merozoite-erythrocyte interaction studies during the erythrocytic phase have revealed some of the specific proteins located on merozoite surface used for interaction with erythrocyte membrane proteins (erythrocyte receptors). The former (invasion ligands) enable parasite adhesion and, during the first steps of invasion, ligand selection associated with the parasite's invasion phenotype (Nery et al., 2006; Iyer et al., 2007). Several invasion phenotypes associated with invasion ligand expression have been described for *P. falciparum*, thereby enabling isolates obtained from infected patients and laboratory strains to be classified into groups (Nery et al., 2006; Ochola-Oyier et al., 2016). Parasite ligand selection has notably been seen in studies involving African populations where differences in invasion ligand expression have been found. For example, slightly higher *eba175* gene expression and lower *eba181* gene expression have been observed when comparing a Ghanaian population to a Senegalese one and possible correlation of this invasion pattern to patients' immunological responses and endemicity levels (Bowyer et al., 2015). Population studies enable analyzing invasion phenotype occurrence and the detection of the most important invasion proteins which could be exploited for developing therapeutic strategies and preventative measures (Bowyer et al., 2015; Ochola-Oyier et al., 2016).

Many studies have shown that a selected invasion phenotype is influenced by the presence/expression of genes encoding parasite ligands, the proteins on erythrocyte surface acting as receptors for them and recognition by a host's immune system (Josling et al., 2015; Diaz et al., 2016; Valmaseda et al., 2017). When completely adapted to *in vitro* conditions, parasites do not have to change because of hostile host conditions (i.e., host immune response and different erythrocyte receptor profile); however, they may change the expression of some virulence-associated genes. Strikingly, Tarr et al. (2018) have described more relaxed transcription in parasite laboratory strains compared to clinical isolates. A slight repression in invasion-related genes has been observed in clinical isolates (Tarr et al., 2018), demonstrated by comparing *pfeba175, pfeba181, pfrh1, pfrh2a, pfrh2b, pfrh4*, and *pfrh5* transcription between six isolates from Ghanaian patients to *P. falciparum* 3D7, Dd2, D10, and HB3 laboratory strains, these genes' lower expression being observed in patient isolates compared to lab strains. Also, a similar behavior has been seen regarding *var* gene expression in parasites infecting naïve patients compared to different isolates from high endemic populations in Africa, as naïve patients have not had the complete repertoire of antibodies to resist parasite infection and have not been able to enforce very strong immune selectivity against erythrocyte membrane protein 1 (*Pf* EMP1) encoded by such *var* genes (Abdi et al., 2016). This has thus suggested that the parasite could have a more relaxed transcription profile and such behavior may occur for *var* genes as well as invasion-related genes like those associated with the *Pf* EBA and *Pf* Rh protein families.

## *P. falciparum* Genetic Structure and Mutation Rate

Along with the parasite's need for adapting to a host's environment, invasion ligand expression depends on its genetic composition and evolution. The *P. falciparum* genome covers fourteen AT-rich chromosomes (reaching 80.6% A-T overall, nearly 80% in encoding regions and almost 90% in non-encoding regions) (Gardner et al., 2002; Hamilton et al., 2016). Such sequence bias has been linked to increased genetic diversity in parasite strains and isolates as it induces insertions and deletions (indels) and unequal cross-over events associated with low-complexity regions as a consequence of polymerase slippage (Lovett, 2004). Such *P. falciparum* genome characteristics promote antigen variability and explain why some surface proteins are present or absent in specific strains.

A genome sequence analysis of the parasite's genetic attributes has been carried out to assess whether genomic composition might influence invasion adaptation; the results showed that the high A-T content in repeat regions of some antigenic proteins, such as merozoite surface proteins (MSP) 1, 2, and 3, might be acting as a strategy for evading a host's immune response, since such high A-T composition was found to be associated with a reduced amount of hydrophobic amino acids and thus a relative increase in hydrophilic ones, these being good targets for antibody responses (Verra and Hughes, 1999). Increased repeat region antigenicity attracts the immune response toward such polymorphic regions, thereby acting as an evasion mechanism.

A mutational analysis comparing six *in vitro-*adapted *P. falciparum* Cambodian field isolates to established *P. falciparum* strains (3D7, HB3, Dd2, and W2) revealed a high G:C to A:T transition rate and low complexity regions in all parasite genomes analyzed, thereby facilitating the appearance of indels. This showed that the parasite has genomic structural malleability, partly enabling its ability to adapt and evolve (Hamilton et al., 2016).

Such genomic changes may explain the parasite's ability to produce a large amount of antigenic variants, thereby enabling it to avoid recognition by a host's immune system; nevertheless, varied invasion gene presence triggered by changes in genomic sequence does not always explain all invasion phenotypes. Evidence suggests that some parasite strains encoding functional copies of invasion ligands can modulate their expression, depending on the availability of receptors on erythrocyte surface (Stubbs et al., 2005). It has also been shown that *P. falciparum* has some flexibility regarding its invasion pathways, allowing it to switch from one invasion phenotype to another, thereby enabling the invasion of erythrocytes having different receptors. This has been experimentally observed for the W2mef strain which predominantly uses sialic acid-dependent ligands, changing its invasion phenotype when forced to invade neuraminidase-treated erythrocytes (sialic acid having been removed by enzymatic treatment) (Stubbs et al., 2005). The parasite's gene expression thus has “on time” transcription related to a specific process during each stage; variations in the parasite's environment (i.e., host conditions such as nutritional and immunological state) may induce transcription regulation changes in phenotype adaptive adjustment-associated genes, like those related to invasion ligands or the switch to gametocytes (Bozdech et al., 2003b; Lu et al., 2017).

Changes in the expression of genes encoding ligand proteins depends on transcriptional regulation, involving a number of specific traits in *P. falciparum* differing from that of other eukaryotes (Deitsch, 2015). The parasite genome's high AT content may explain the shortage of transcriptional regulation mechanisms in *P. falciparum* when compared to other eukaryotic organisms (Coulson et al., 2004). Such genomic composition may have hampered the discovery of transcriptional regulatory pathways in *P. falciparum*, requiring the use of experimental tests as comparative genomics in the search for these elusive mechanisms (Balaji et al., 2005). Nevertheless, the development of technologies such as microarrays, chromatin hybridization and immunoprecipitation, and sequencing assays has led to the discovery of relationships between promoter sequences and transcription factors with protein expression (Josling et al., 2015; Santos et al., 2017; Filarsky et al., 2018; Fraschka et al., 2018).

## Transcriptional Changes Modulating Invasion-Associated Gene Expression

It should be stressed that *P. falciparum* gene structure is similar to that of other eukaryotic genes, consisting of open reading frames (ORF), having intergenic regions and promoter sequences. Like other eukaryotes, the parasite's mRNA is processed in the nucleus and translated in the cytoplasm; the transcription machinery is partly responsible for regulating expression, being modulated in response to environmental stimuli like temperature, glucose levels and stress factors (Hughes et al., 2010). The parasite's intra-erythrocytic cycle thereby involves transcription following an ordered pathway, connected to the parasite's needs during each stage in the cycle (Bozdech et al., 2003a). Transcription is thus governed by a balance between regulatory elements in the DNA (elements in *cis*) and transcription factors (elements in *trans*) (Gissot et al., 2005). Most genes are transcribed during the trophozoite stage, being related to several parasite functions including metabolism, intracellular protein transport, and protein catabolism (Lu et al., 2017). However, specific genes related to merozoite entry to erythrocytes, such as those encoding the proteins associated with the parasite's rhoptries and micronemes, are expressed during the schizont stage (Lu et al., 2017).

Even though *P. falciparum* has comparatively few transcription factors, several regulatory elements and protein families have been discovered (Balaji et al., 2005). Many of the parasite's *cis*-elements which regulate gene expression have been identified after in-depth bioinformatics analysis of the parasite genome, microarray gene expression data and RNA-seq (Gunasekera et al., 2007; Jurgelenaite et al., 2009). Such *cis*-elements include discrete sequence motifs which are over-represented in promoters and regulatory regions (Jurgelenaite et al., 2009). These motifs have been found to be linked to specific transcription factors during every parasite stage, such as those belonging to the apicomplexan activator protein-2 (ApiAP2 or apetala2) family (Campbell et al., 2010).

The ApiAP2 family of transcription factors is a group of proteins which binds to specific upstream motifs and positively or negatively regulates target gene transcription in plants (Jofuku, 1994; Okamuro et al., 1997). These *apiap2* genes' orthologous genes have been found in *P. falciparum*, having similar structure and regulatory functions (Balaji et al., 2005; Painter et al., 2011; Tuteja et al., 2011). Balaji et al. (2005) reported these *apiap2* orthologs in *P. falciparum* as the family of transcription factors (*Pf* AP2), describing 27 proteins associated with the transcription of specific genes in *P. falciparum* during precise phases of the parasite's life cycle (Balaji et al., 2005). Twenty of these proteins are expressed during the intra-erythrocytic cycle and some of them regulate invasion protein expression, specifically, those called *Pf* AP2-Invasion (*Pf* AP2-I) acting on genes associated with invasion by binding to the NGGTGCA upstream motif (Santos et al., 2017).

The mechanism by which *Pf* AP2-I transcription factor regulates invasion-related gene expression has been studied by Santos et al. (2017). The CRISPR/Cas9 gene-editing system was used to produce a mutated strain of the parasite carrying a deletion in the ATGCA *Pf* AP2-I-binding motif upstream of the *P. falciparum* merozoite surface protein gene 5 (*pfmsp5*) to evaluate the motif's importance in gene transcription (Santos et al., 2017). Decreased *pfmsp5* expression was observed in the mutant strain, thereby confirming the motif's relevance for transcriptional regulation. It is worth noting that there was no significant reduction in *Pfrh4* expression, suggesting a different regulatory mechanism for this gene (Santos et al., 2017). Interestingly, using GEO2R software (https://www.ncbi.nlm.nih.gov/geo/geo2r/) for reanalysing Santos et al. (2017) expression data (GEO: GSE77807, available in the Gene Expression Omnibus (GEO) database), led to finding increased expression of invasion-related genes, such as reticulocyte binding protein 2 homolog b (*pfrh2b*) and *Pf* EMP1-associated *var* gene in the mutated strain compared to the non-mutated one. The authors reported no significant changes regarding *pfeba140, pfeba181, pfeba175, pfrh1, pfrh2a, pfrh2b and pfrh5* expression (Santos et al., 2017, Supplementary data). This could suggest a compensatory mechanism regarding invasion ligand expression which could be regulated by a different motif linked to *Pf* AP2-I, or that other transcription factors are also involved in regulating these proteins; however, further studies are needed.

## *P. falciparum* Transcription Factors Could Act by Forming Complexes

Gene-expression regulation is associated in most eukaryote organisms with protein complex formation. The interaction of the *Pf* AP2 protein family with other transcription factors and the formation of protein complexes is thus expected; Josling et al. (2015) have shown that *P. falciparum* bromodomain protein 1 (*Pf* BDP1) is a *Pf* AP2 coactivator regulating transcription during invasion, possibly as a secondary effect after complex formation between these two proteins (Josling et al., 2015; Santos et al., 2017). *Pfbdp1* gene silencing reduces invasion, evidenced by decreased merozoite reorientation and invagination, accompanied by the downregulation of some genes linked to invasion (i.e., *Rh2a, Rh4, eba181*) and with the parasite's motility mechanisms [i.e., glideosome-associated protein 40 (*gap40*), rhomboid protease ROM4 (*rom4*), glideosome associated protein with multiple membrane spans 1 (*gapm1*) and glideosome-associated protein with multiple membrane spans 2 (*gapm2*)] (Josling et al., 2015).

It has been described that *Pf* AP2-I specifically interacts with *Pf* BDP1 which is associated with the up-regulation of key genes during invasion, such as *msp1* and apical membrane antigen 1 (*ama1*) (Josling et al., 2015; Santos et al., 2017). However, such correlation was weaker for other genes expressed early on during merozoite invasion, such as *pfeba175 pfeba140*) (Josling et al., 2015). These results suggested the presence of other proteins acting during these genes' transcription regulation that have not yet been described (co-activators being present according to step-by-step or “in time” parasite invasion).

Consequently, it can be foreseen that the parasite's transcription factors form transcription activation complexes and are negatively regulated by other proteins until the signaling which induces transcription activation occurs. Expanding on this argument, a family of proteins called acetylation lowers binding affinity (Alba) has repressive activity during transcription, is conserved in other eukaryotes, binds both DNA and RNA and could play a role in parasite transcription regulation (Bell et al., 2002; Chêne et al., 2012). Four ortholog genes encoding Alba proteins have been described in *P. falciparum* (*Pf* Alba-1-4), binding DNA in a similar way to that of orthologs found in other eukaryotes (Chêne et al., 2012). However, whilst Albas have been reported to bind to DNA in archaea without sequence specificity, *Pf* Alba-1, *Pf* Alba-2, and *Pf* Alba-4 do have it; such sequence specificity could be related to the Alba protein's function in the parasite (Wardleworth et al., 2002; Chêne et al., 2012). Alba proteins are inactivated by deacetylation of a lysine residue (Lys16), catalyzed by sirtuin 2 (Sir2), thereby affecting the protein's interaction with DNA and enabling the action of other transcription factors (Bell et al., 2002; Wardleworth et al., 2002).

Molecular analysis of transcription and protein expression has shown the importance of *Pf* Alba-1 in mRNA homeostasis maintenance in trophozoites as it represses the translation of some invasion genes [e.g., *P. falciparum* high molecular weight rhoptry protein 3 (*Pf* RhopH3) and *P. falciparum* calcium-dependent protein kinase 1 (*Pf* CDPK1)] until its transcription its needed (late schizont stage), suggesting fine-tuning of translation time (Vembar et al., 2015). The *Pf* Alba-1 protein could form a complex with *Pf* Alba-2 and *Pf* Alba-4 and might also interact with *Pf* AP2-I proteins and, indirectly, with *Pf* BDP1 through the already known *Pf* AP2-I and *Pf* Alba-4 interaction, thereby indicating that *Pf* Alba proteins may be involved in the transcriptional regulation of invasion proteins (Josling et al., 2015; Vembar et al., 2015).

## Other *P. falciparum* Transcription Factors

*Pf* AP2 proteins have been specified as playing the most important role in transcription regulation; however, other transcription factors are expressed in eukaryotes having orthologs in *P. falciparum* and seem to be carrying out the same function, i.e., the myeloblastosis (Myb) transcription factor, the pre-initiation complex (PIC) and nuclear factors related to the high-mobility-group (HMG) box (Tuteja et al., 2011; Goyal et al., 2012). Myb-related proteins bind DNA in a sequence-specific manner in other eukaryotes and regulate the expression of genes associated with growth and differentiation (Boschet et al., 2004). *Pf* Myb1 has been found to be involved in DNA/protein interaction during the trophozoite stage and is important in parasite transition from trophozoite to schizont stage since it regulates the transcription of genes which are relevant for the cell cycle, such as *Pf* Pk5 (cdc2-related cyclin-dependent kinase (CDK) acting as cell cycle control and progression regulator), histone 3 (H3), and histone 2A (H2A) (Gissot et al., 2005).

The pre-initiation complex (PIC) comprise the *P. falciparum* TATA box binding protein (*Pf* TBP) and the transcription factor IIE (*Pf* TFIIE), coordinating transcriptional initiation, promoting loop formation enabling interaction with other transcription factors and favoring nucleosome assembly (Gopalakrishnan et al., 2009). Chromatin immunoprecipitation (ChIP) assays have shown that these proteins are located in the promoter sequences of genes expressed during ring (e.g., *hrp3* and *sbp1*) and trophozoite stages (e.g., *msp1* and *rhoph3*) (Gopalakrishnan et al., 2009). High mobility group box proteins (HMBP) binding DNA and regulating transcription and chromatin architecture in other eukaryotes (Malarkey and Churchill, 2012) also have orthologs in *P. falciparum* (Briquet et al., 2006).

*Pf* HMGB1 and *Pf* HMGB2 have specificity for DNA, are located in the trophozoite and schizont nuclei and are involved in DNA bending, modifying the nucleosome structure and inducing transcriptional changes (Briquet et al., 2006). Immunofluorescence assay subcellular localization of *Pf* HMGB proteins on asexual (trophozoites, schizonts) and sexual (gametes) parasite forms has shown that both proteins are expressed in the nucleus during intra-erythrocytic cycle stages and that *Pf* HMGB2 was also present in gametocytes cytoplasm. This led to the conclusion that *Pf* HMGB1 could be associated with proliferation whilst *Pf* HMGB2 might be involved in the parasite's sexual differentiation (Briquet et al., 2006).

Recent evidence using the GCACTTTTATTGCA motif for DNA pull-down assays has suggested a relationship between AP2-I, *Pf* HMBG3 recruitment and other chromatin factors (such as *Pf* BDP1, 2, and 3) for targeting gene regulation promoter sequences (Toenhake et al., 2018). The GCACTTTTATTGCA motif has also been found in invasion-related gene promoters such as *msp2, msp4, roph3* (Santos et al., 2017); such findings suggest a relationship between *Pf* HMBG3 and some invasion-related gene expression.

## Epigenetic Modifications Regulate Loci Transcriptional Status

Another significant aspect of transcription variation is related to the epigenetic changes associated with a host's environment which could influence invasion patterns (Fraschka et al., 2018). Most of the parasite's chromatin remains in euchromatic state and some specialized regions having a redundant function during invasion are in heterochromatic state (Duffy et al., 2012). These heterochromatic regions functionally regulate gene expression via epigenetic changes, keeping these genes transcriptionally active or silenced when required (Voss et al., 2014; Ay et al., 2015; Duraisingh and Skillman, 2018; Fraschka et al., 2018).

It is worth analyzing the parasite's nucleosome structure as chromatin state plays a role in transcriptional status; it consists of a DNA strain wrapped around a complex of H3, H4, H2A, and H2B histones (Miao et al., 2006). H3, H4, and H2A are predominantly acetylated by histone acetyltransferases (HAT) in trophozoites and schizonts during the intra-erythrocyte cycle; this modification is primarily associated with nucleosome unpacking and opening (Miao et al., 2006). ChIP-qPCR assays of the acetylated lysine 9 in histone 3 (H3K9ac) in the *P. falciparum* NF54 strain have shown that this mark is abundant during schizont stage and is linked to the induction of gene expression during this stage (Salcedo-Amaya et al., 2009). It has also been found that the mark is added by histone *P. falciparum* acetyltransferase general control nonderepressible 5 (*Pf* GCN5) (Cui et al., 2007; Salcedo-Amaya et al., 2009).

Nucleosome modifications (opening and closing) are directly correlated with transcription activation and the amount of transcriptional activity occurring during the intra-erythrocytic cycle (Salcedo-Amaya et al., 2009). It has been discovered that most transcription happens during the trophozoite stage when a peak in nucleosome opening occurs and most genes are transcribed (Ay et al., 2015). Nucleosomes close up when the parasite reaches mature schizont stage, thereby halting transcription; such stage contains fresh merozoite which will exit and infect other erythrocytes (Ay et al., 2015). Parasite phenotype variations might then be correlated with transcriptional regulation and the parasite's chromatin remodeling by epigenetic machinery, these being changes related to the parasite's environment (Voss et al., 2014; Fraschka et al., 2018; Rono et al., 2018).

Heterochromatin protein 1 (HP1) is important for chromatin remodeling and has been previously described in eukaryote organisms as being related to heterochromatin structure and gene silencing or activation; it has an ortholog in the parasite [*P. falciparum* heterochromatin protein 1 (*Pf* HP1)] (Lomberk et al., 2006; Brancucci et al., 2014; Canzio et al., 2014). *Pf* HP1 binds to H3K9me2/3 and mediates heterochromatin formation by recruiting chromatin remodeling factors which compact and confine silent portions of the genome to the inner side of the nuclear envelope (Lomberk et al., 2006; Brancucci et al., 2014; Canzio et al., 2014). H3K9me2/3 marks of constitutive heterochromatin have also been linked to *Pf* AP2-G-mediated transcriptional regulation in gametocytes (Rovira-Graells et al., 2012).

It has been demonstrated that *Pf* HP1 has strain-specific occupancy in heterochromatic regions for its regulation function, as shown by ChIP-seq analysis of four *P. falciparum* strains (NF54, NF135, 3D7 and Pf2004), and plays a role in silencing development genes related to parasite differentiation into sexual forms, regulating *PfAP2-G* gene activation and maintaining the parasite during its asexual stage (Rovira-Graells et al., 2012; Fraschka et al., 2018). Some heterochromatic regions have also been shown to be long-lasting and hereditable, thereby explaining the variable expression observed in some *P. falciparum* strain genes and its persistence in isolate populations (Fraschka et al., 2018).

*Pf* HP1's importance for asexual stage maintenance may imply an indirect relationship between this regulatory protein and merozoite invasion ligands. Signaling pathway components should thus link the sensing of environmental changes with *Pf* HP1/H3K9me3-mediated gene silencing and heterochromatin formation [as seen in parasite nutrient deprivation and the relationship between parasite gametocyte production and *Pf* HP1 (Brancucci et al., 2014; Delves et al., 2016)]. A later study described *Pf* HP1 correlation with its antagonist (perinuclear protein *P. falciparum* gametocyte development 1 - *Pf* GDV1) for gametocytogenesis (Filarsky et al., 2018). This relationship is important for the parasite's sexual differentiation since *Pf* GDV1 triggers *Pf* HP1 removal from heterochromatin, thereby enabling the parasite's sexual differentiation (Filarsky et al., 2018). The same study's comparative transcriptome analysis of a parasite line having conditional *Pf* GDV1 expression compared to the *P. falciparum* wild type F12 strain showed changes in some invasion ligand gene expression (Filarsky et al., 2018). For example, a 71.44% decrease in *rh5* gene expression was seen during schizont stage and a 14.28% increase in *eba175* expression in the knock down strain compared to the wild type (Filarsky et al., 2018). Such data suggested an indirect correlation between these transcription regulators and invasion ligands expression (meriting further study).

It is also known that when eukaryote organisms are nutrient-deprived (i.e., apicomplexan *Toxoplasma*) they activate signaling pathways for survival, related to the target of rapamycin (TOR) and autophagy pathways (Ghosh et al., 2012). *Plasmodium* does not have a TOR protein ortholog, but does have orthologs for some autophagous genes (*Atg*), like *Atg8, Atg7*, and *Atg3*, and may share signaling pathways for nutrient starvation or deprivation (Hain and Bosch, 2013; Cervantes et al., 2014). Inhibitor compounds (Torin1 and Torin2) of the mechanistic target of the rapamycin (mTOR) pathway have a gametocytocidal effect, as has been described for 3D7, HB3, and Dd2 strains of *P. falciparum* (Sun et al., 2014). This could suggest an indirect relationship between the autophagy pathway and *Pf* HP1/H3K9me3-mediated gene expression regulation (though experimental support for such relationship is still lacking). Such findings suggest a relationship between parasite ligand-erythrocyte receptor interaction with the activation of signaling pathways reinforcing the transcription and expression of these ligands mediated by *Pf* PH1/H3K9me3 epigenetic remodeling, but this hypothesis requires confirmation.

## Host Conditions Could Induce Parasite Phenotype Changes

The parasite must initially face two “big barriers” for successful erythrocyte invasion, almost simultaneously. One such obstacle is the host's immune response, repressing host antibody recognition, as the parasite's cell membrane and its surface proteins are exposed and susceptible to being recognized in the bloodstream (Persson et al., 2008). The other barrier concerns erythrocyte receptor accessibility, which could change, depending on the host's origin and genetic characteristics (Leffler et al., 2017). This means that both sequence polymorphism and the parasite's differential adaptive expression of invasion ligands are needed to overcome a host's invasion barriers (Persson et al., 2008; Leffler et al., 2017). In other words, the parasite's phenotype is influenced by the host's immune response and proteins present on the erythrocyte surface.

On the other hand, amongst parasite mechanisms to deal with the environmental changes (such as temperature shifts and high- or low-transmission environments), aleatory gene expression switches conferring a better adaptation to fluctuating environments might be playing an important role, as has been described in other eukaryotes (Acar et al., 2008; Rovira-Graells et al., 2012; Rono et al., 2018). By selecting ones over others in a stochastic pattern, phenotypes that confer an invasion advantage will prevail, allowing parasite survival (Rovira-Graells et al., 2012). The real contribution of the stochastic versus the adaptive mechanism in the parasite's phenotypic switching remains to be determined, but both mechanisms would appear as important ways to cope with erythrocyte receptor variations and to avoid immune recognition (Stubbs et al., 2005; Wright and Rayner, 2014).

Immunity against malaria is induced when parasites or proteins shed to the extracellular space are exposed to host immune recognition. Some highly expressed *P. falciparum* proteins, like EMP1, are recognized by the host's immune system, thereby inducing antibody production (Turner et al., 2015; Abdi et al., 2016). Certain host populations' immune-response traits (e.g., the prevalence of some human leukocyte antigen alleles) have led different parasite strains to preferentially express some invasion proteins rather than others (Nery et al., 2006; Bowyer et al., 2015; Ochola-Oyier et al., 2016). This phenomenon has also been linked to the appearance of chronic infection in some patients (Abdi et al., 2016). It can also be concluded that geographical isolation and constant exposure to the host's immune system is critical in relation to the emergence of differences in isolates from geographically-distant regions (Mensah-Brown et al., 2015; Ochola-Oyier et al., 2016; Rono et al., 2018).

## The Host's Immune Response Can Induce Changes in Invasion Ligand Expression

An immune response to the parasite depends on a particular host's immunological state and the amount of infections suffered (Schofield and Mueller, 2006). Specifically, merozoite surface antigen exposure to immunological attack during the erythrocyte phase of infection highlights the importance of the speed of erythrocyte invasion as one of the main parasite mechanisms for avoiding its neutralization by the complement system and host antibodies. However, this is not the only mechanism used by the parasite to ensure its survival (Wright and Rayner, 2014).

Most pathogens adopt two strategies for modifying their surface protein expression to bypass detection by a host's immune system: changing some antigens' expression in an “on-off” fashion and by expressing different forms of a particular antigen (Deitsch et al., 2009). Most *P. falciparum* strain has sequence differences and variable expression levels regarding exposed antigens to avoid antibody recognition (Schofield and Mueller, 2006; Wu et al., 2014; Xia et al., 2018). These mechanisms enable a parasite's immune evasion and the emergence of clinical immunity, manifest by the absence of clinical symptoms. A parasite's chronic exposure to a host facilitates its adaptation mechanisms, decreasing parasitaemia, changing its invasion phenotype and modulating erythrocyte invasion ligand expression, making itself invisible to a host's immune system (Schofield and Mueller, 2006; Persson et al., 2008; Xia et al., 2018); immune evasion is therefore crucial for parasite survival, establishing chronic infection and transmission.

*P. falciparum has* developed a strategy favoring the modulation of critical proteins' expression; *var* gene family expression is an example of such adaptive behavior, having clonal variation and gene expression regulation linked to dynamic remodeling of chromatin, enabling “on-off” modulation regarding these genes' expression due to its subtelomeric location and *Pf* Sir2 repression function (Freitas-Junior et al., 2005).

The parasite must thus modulate the expression of its proteins; this was seen in parasites having higher *Pf* EMP1 expression when analyzing controlled infection of Kenyan volunteers having low to moderate exposure to *P. falciparum* (Abdi et al., 2017). Parasites having high *Pf* EMP1 expression was observed in individuals having had little exposure *P. falciparum* malaria; by contrast, individuals having moderate to high exposure had more antibodies against this protein; parasite *Pf* EMP1 expression was lowered, reaffirming the hypothesis concerning immune escape by modulating invasion proteins (Abdi et al., 2017). A study by Rono et al. (2018), comparing the transcriptome of 96 parasite isolates from patients living in regions of Africa having variable malarial incidence, demonstrated that parasites modulate the expression of their invasion proteins when parasitaemia is low to ensure their survival or change their differentiation programming from asexual to sexual stage, as seen in regions having low malarial incidence, where increased gametocyte production has been reported (Rono et al., 2018).

The *Pf* EBA and *Pf* Rh protein families can also be included in this group of modulated proteins playing a role concerning phenotypic variations, as they have redundant functions thereby helping the parasite to avoid the host's immune system as there is some variability in antibody production against the parasite's invasion ligands (Tijani et al., 2018). Such variability has been described in a longitudinal study which analyzed 156 Nigerian's immune response; different and fluctuating immune responses against *Pf* EBA175 and *Pf* Rh2 antigens were found. It described higher antibody levels against these two antigens in people having suffered more infecting events compared to those having had less episodes (Tijani et al., 2018). This data substantiates the fact that the parasite must adapt to different host environments, even in populations from the same geographical areas.

## Erythrocyte Surface Receptors Could be Related to Predominant Parasite Invasion Ligand Expression

Moving on now to consider the relationship between parasite invasion phenotype and erythrocyte surface receptors, the evidence regarding human and *Plasmodium* co-evolution should be considered. The parasite exerts selective pressure on human hosts, causing changes in erythrocyte surface protein expression and polymorphism fixation conferring infection resistance (Ko et al., 2011). Invasion-related phenotype heterogenicity may be further associated with the proteins in the erythrocyte's cytoplasmic membrane, some of which are related to an individual's blood group (Baum et al., 2003; Theron et al., 2018).

Thirty-four blood groups related to surface antigens on the erythrocyte cytoplasmic membrane have been characterized by the International Society of Blood Transfusion (ISBT) (Reid and Lomas-Francis, 2004). ABO is the best known of them; it is associated with 4 blood groups (A, B, AB, O) related to the *ABO* gene encoding A alleles (encoding 3-α-N-acetylgalactosaminyltransferase (GalNAc) which transfers N-acetylgalactosamine from the uridine diphosphate (UDP)-N-acetylgalactosamine substrate to the H antigen, which contains a fucosylated galactosyl residue, thus forming the A antigen), B alleles (encoding 3-α-galactosyltransferase (Gal) adding a D-galactose from UDP-galactose to the H antigen in the fucosylated galactosyl residue, thus creating the B antigen) and a null allele producing a non-functional enzyme to produce the O group (Reid and Lomas-Francis, 2004; Daniels, 2005). A protective effect for the O group against severe malaria (associated with thrombosis and microvascular ischemia) has been reported: this has been related to lower *P. falciparum* rosetting induction in O group erythrocytes compared to the A, B, and AB groups (Rowe et al., 2009). Nevertheless, the O blood group occurs most frequently in endemic areas; a study of 40 healthy donors from the United Kingdom has shown preferential *P. falciparum in vitro* invasion of O group erythrocytes, suggesting parasite adaptation to the most abundant blood group (Theron et al., 2018).

The MSN blood group has been the most studied to date in attempts to understand the invasion-relationship between the parasite and erythrocytes; it has been shown that parasite pressure induces variations in these blood group antigens (Dolan et al., 1994; Li et al., 2012). This specific blood group (i.e., M, N, and S antigens), which has been directly related to merozoite interaction with erythrocytes during invasion, is related to the *GYPA* and *GYPB* genes encoding glycophorin A (GPA) and glycophorin B (GPB) integral, single span, membrane proteins (Reid and Lomas-Francis, 2004). It is known that treatment with trypsin cleaves GPA into its residues 31 and 39 and that treatment with chymotrypsin cleaves GPB at residue 32; such knowledge has enabled analysis of the interaction between parasite ligands and erythrocyte receptors (Reid and Lomas-Francis, 2004; Tham et al., 2012). It has also been described that erythrocytes having mutations or deletions in these antigens are related to reduced merozoite invasion of host cells [i.e., *GYPA* (En(a-)] or *GYPB* (S-s-) deletion) because these proteins are implicated in EBA175 (GPA) and EBL1 (GPB) ligand binding (Dolan et al., 1994; Li et al., 2012).

Human glycophorin genes' rapid evolution has been associated with an erythrocyte infection evasion mechanism, thereby correlating with malarial prevalence in affected human populations (Wang et al., 2003). Glycophorin gene sequence diversity analysis in populations living in African malaria-endemic regions has shown allelic variability acquired through human evolution in such receptors, thereby validating these findings and the fact that this might put selective pressure on parasites (Leffler et al., 2017). A study of *GPA, GPB*, and *GPE* genes from 282 unrelated individuals native to 15 African group having different ethnicity, representing genetically diverse populations and geographical origins having different malarial incidence in Africa, has shown high nucleotide diversity regarding the three genes in all populations (Ko et al., 2011). Other studies have reported glycophorin gene copy number variations in a Sub-Saharan population, describing hybrid gene structures (*GYPB-A* and *GYPE-A* hybrids) and *GYPB-A* hybrid correlation (i.e., Dantu, a particular gene rearrangement); this has previously been described as a malaria erythrocyte invasion protector antigen, as assessed by *in vitro* invasion assays (Field et al., 1994; Leffler et al., 2017). The host's defense strategy also includes modifications in glycophorin's O-sialoglycan-rich region, inducing structural changes which alter parasite ligand-binding affinity to these receptors without affecting protein function (Jentoft, 1990; Ko et al., 2011).

The Gerbich blood group is another blood group which has been studied regarding its relationship with malaria; it has been associated with the glycophorin C protein (GPC), encoded by the *GYPC* gene (Reid and Lomas-Francis, 2004). GPC is cleaved by trypsin at residue 48 and is associated with parasite ligand EBA140; changes in this erythrocyte antigen affect merozoite attachment and some of these changes have been correlated with Yus (lacking *GYPC* gene exon two) and Leach blood groups (lacking *GYPC* exons 2, 3, and 4) (Lobo et al., 2003; Reid and Lomas-Francis, 2004).

The Duffy blood group, another important blood group in invasion by other *Plasmodium* species, such as *P. vivax*, has been thoroughly described; it is also known as the Duffy antigen receptor for chemokines (DARC), referring to antigens encoded by the *FY* gene, having alleles *FY*^*a*^ and *FY*^*b*^, which are related to Fy^(a^+^*b*^−^)^, Fy^(a^−^*b*^+^)^, Fy^(a^+^*b*^+^)^, and Fy ^(a^−^*b*^−^)^ antigens. Fy ^(a^−^*b*^−^)^ antigen results from mutations in the *FY*^*b*^ allele promoter site thereby hampering globin transcription factor 1 (GATA1) binding (Miller et al., 1976; Tournamille et al., 1995; Daniels, 2005). Fy ^(a^−^*b*^−^)^ is more prevalent in African and African-American populations and correlates with protection against *P. vivax* and *P. knowlesi* because it impedes the parasite's Duffy binding protein's (DBP) interaction with DARC; it is important during invasion for merozoite attachment and tight junction formation between merozoite and erythrocytes (Miller et al., 1976; Adams et al., 1992). However, recent reports have described *P. vivax* transmission in an Fy ^(a^−^*b*^−^)^ population in several parts of Africa, such as Kenya, Cameroon, Mali, Equatorial Guinea and Madagascar, indicating the parasite's possible evolutionary adaptation to changes in erythrocyte surface receptors (Ryan et al., 2006; Ménard et al., 2010; Mendes et al., 2011; Niangaly et al., 2017; Russo et al., 2017).

It can thus be thought that the parasite senses the host surface membrane probably with two purposes: to attach to and invade host cells, and for reinvasion during subsequent cycles. Epigenetic changes must be made regarding the latter to express suitable alternative erythrocyte binding ligands promoting successful invasion.

Such epigenetic adaptive changes (occurring due to variations in erythrocyte surface protein availability) could appear during both ring and trophozoite stages and are associated with chromatin changes and transcriptional regulation by transcription factors (Stubbs et al., 2005; Painter et al., 2011, 2018; Coleman et al., 2012; Ay et al., 2014). Eukaryote adaptive mechanisms may be associated with enzyme removal by mutational mechanisms or signaling pathway alterations (Claessens et al., 2017). It could be thought (regarding the parasite) that there is an association between ligand-receptor binding failure and changes in signaling pathways related to invasion adaptation mechanisms. *P. falciparum* w2mef strain adaptation phenomena for invading neuraminidase-treated erythrocytes could be an example of this behavior (Stubbs et al., 2005).

Some parasite ligands bind to glycophorins on erythrocyte membrane; the glycophorin A sialoglycoprotein being the most abundant; it acts as a receptor for the *Pf* EBA175 ligand. An increase in merozoite calcium concentration is needed for *Pf* EBA175 secretion from microneme merozoite organelles (Aniweh et al., 2016). It is now known that *Pf* Rh1 and *Pf* Rh2b binding to their erythrocyte receptors increases parasite calcium levels and that blocking this interaction (using monoclonal antibodies) inhibits calcium release and also *Pf* EBA175 secretion (Aniweh et al., 2016). Calcium release into erythrocytes also requires an effective ligand-receptor interaction (Volz et al., 2016). This strongly suggests that an increase in erythrocyte calcium concentration could obey parasite calcium-associated signaling changes related to ligand-receptor interactions.

**Figure 1:**
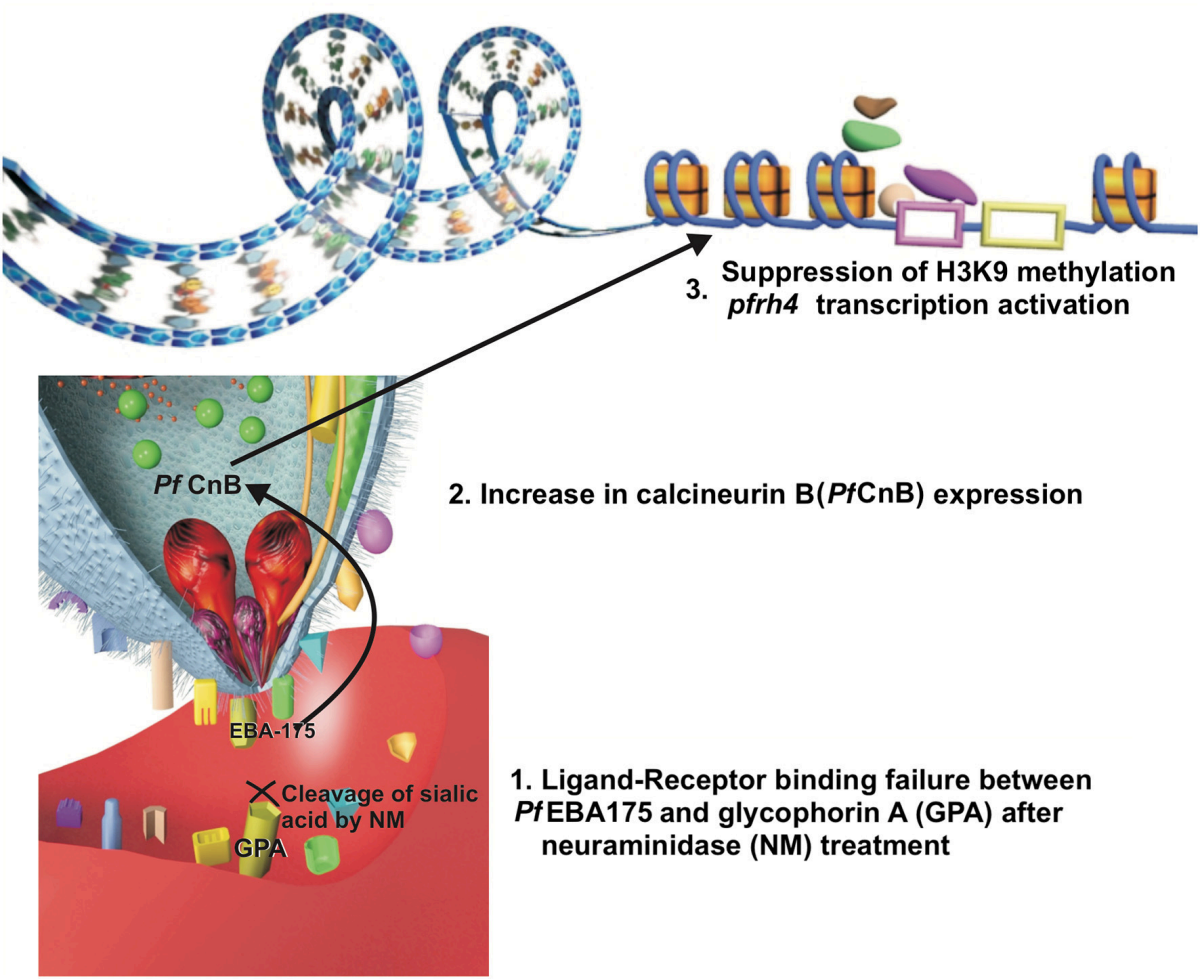
A hypothesis of the relation between ligand-receptor binding failure and a signal transduction pathway. The ligand-receptor binding failure between *Pf* EBA175 and glycophorin A may induce, inside the merozoite, an increase in the expression of calcineurin B (*Pf* CnB) which could be associated (by its possible relation with class I histone deacetylase inhibitors) with suppression of H3K9-methylation, allowing *Pf* Rh4 transcription.

Neuraminidase erythrocyte treatment disrupts the *Pf* EBA protein-erythrocyte interaction, including *Pf* EBA175 (Cowman et al., 2017). It is worth noting that enzymatic treatment of erythrocytes decreases transgenic *P. falciparum* D10 parasite invasion capability, having conditional knockdown for the calcineurin B (*pfcnb*) gene (Paul et al., 2015). Conversely, *Pf* CnB overexpression increases these parasites' capability for invading enzyme-treated erythrocytes, thereby highlighting *Pf* CnB regulation of host cell attachment (Paul et al., 2015). An indirect correlation between *Pf* EBA175-glycophorin and *Pf* CnB interaction could be expected and might be associated with a signaling pathway facilitating alternative ligand expression (Figure 1); studies of such specific interaction could lead to obtaining significant information about the parasite's adaptive behavior.

It has also been described that the parasite could spontaneously switch its invasion phenotype from sialic acid-dependent to sialic acid-independent when it is cultured in shaking conditions, as has been seen for the *P. falciparum* Dd2 and W2mef strains (Awandare et al., 2018). It has also been reported that parasites cultured in shaking conditions increase their dependence on calcineurin for erythrocyte reinvasion, thus highlighting its importance in invasion switching and, to some extent, confirming its role in signaling for parasite adaptation (Paul et al., 2015).

Histone acetylation may be a mechanism enabling repressed alternative gene transcription. This could apply to *Pfrh4* gene expression, previously described as being epigenetically regulated by its relationship with the H3K9 mark and the repression of this gene expression when this mark is methylated (H3K9me3) (Jiang et al., 2010; Coleman et al., 2012). The relationship of calcineurin B expression with class I histone deacetylase inhibitors (associated with H3K9 methylation suppression) has been described in neurons related to changes in the expression of genes conferring a neuroprotective effect (Huang et al., 2011; Chou et al., 2014; Takamatsu et al., 2017). *Pf* CnB may have similar behavior, being related to a pathway inducing changes in histone H3K9 related to *Pfrh4* thereby enabling the exposure of the gene sequence and its regulatory sequences to the transcription factors, facilitating this gene's transcription and the expression of this alternative ligand protein. Analyzing such specific calcineurin-mediated alternative-ligand expression signaling pathway could provide information facilitating the development of antimalarial drugs (i.e., as it has not been completely studied in the parasite).

## Conclusions

**Figure 2:**
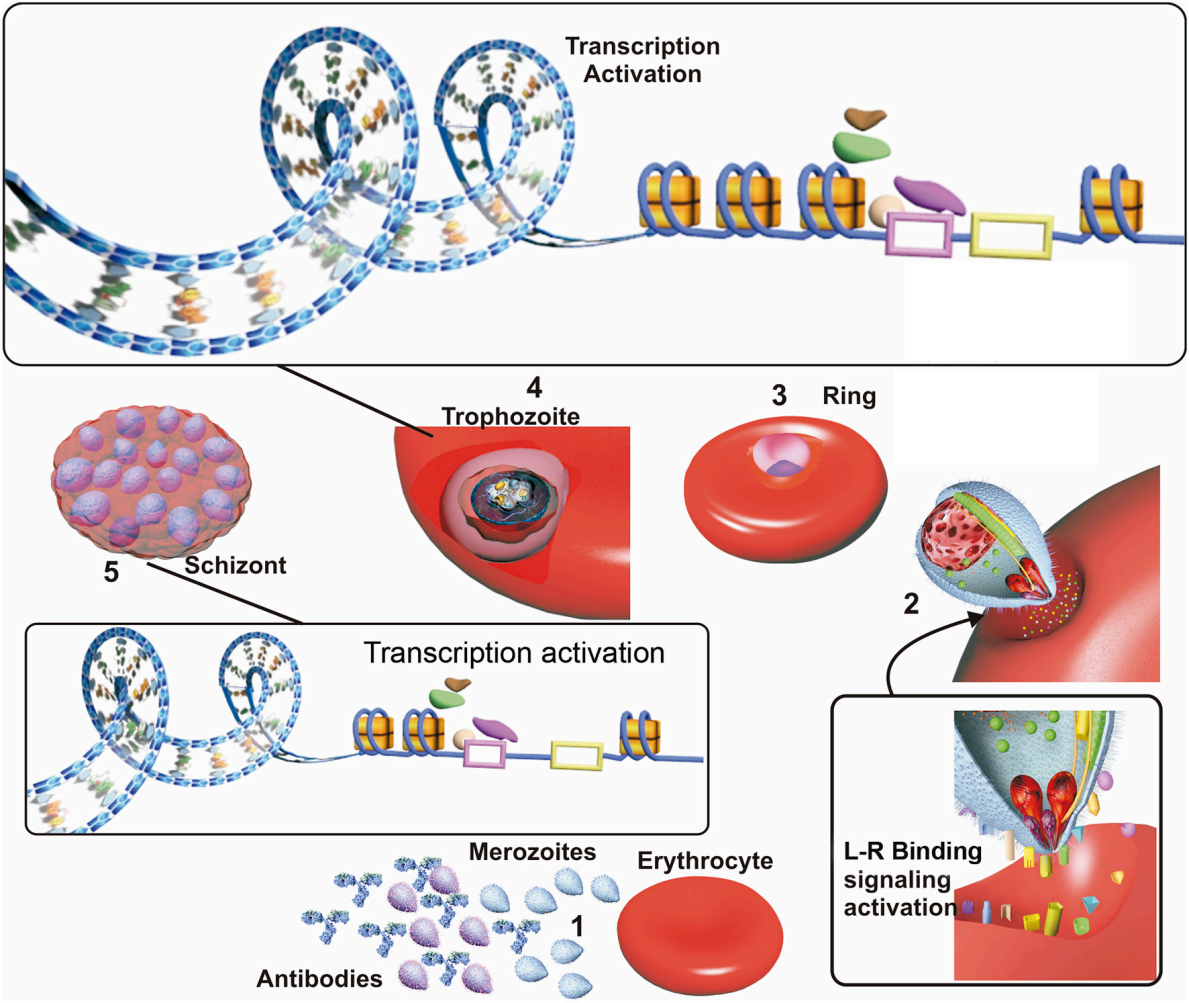
Factors affecting invasion. (1) Merozoites in the blood stream when the erythrocyte cycle takes place are exposed to host antibodies which recognize and inactivate merozoite having higher surface antigen expression in patients exposed to malaria. (2) Merozoites which are not inactivated by antibodies use their surface ligand proteins for erythrocyte sensing and attachment, thereby initiating invasion. Ligand/receptor (L-R) binding may activate signaling pathways, improving these ligands transcription and triggering their selection. (3) The parasite develops to ring state and then trophozoite stage. (4) Most transcription take place during the trophozoite stage; this begins with the opening of the nucleosomes and recruitment of transcription factors acting as transcription activators or repressors, which are related to “in time” expression. (5) The parasite develops into a schizont, invasion genes transcription occurs since transcription repression of them becomes halted. Receptor-ligand interactions between the merozoite and the erythrocyte could be related to the selection of the genes expressed during this phase.

The *P. falciparum* parasite must sense a targeted host's nutritional status, evade its immune response and bind to available erythrocyte receptors for efficient invasion and development (Bowyer et al., 2015; Mancio-Silva et al., 2017) (Figure 2). Effective invasion means that the parasite must adopt some adaptive modifications, such as changes in the parasite's surface invasion protein expression. The evolution of a host's defense, involving an immune response against *P. falciparum* invasion proteins and changes in erythrocyte membrane surface receptors, are also crucial during malarial infection.

Many adaptive changes have co-evolved in the parasite and the human host, conferring the plasticity needed for parasite invasion of erythrocyte populations (Ko et al., 2011; Rono et al., 2018). Epigenetic and transcriptional regulatory mechanisms modulate invasion gene transcription during the co-evolutionary adaptive changes taking place in the parasite, especially in *P. falciparum* with its peculiar genomic composition leading to both transcriptional and genomic sequence changes (Coleman et al., 2012; Wright and Rayner, 2014). A host evolves to recognize and eliminate the parasite through its immune system and prevent merozoite binding to erythrocytes by structural changes in its erythrocyte receptors or by repressing these receptors' expression (e.g., Duffy null allele, or conformational changes found in glycophorin proteins on erythrocytes from some African populations). Some modifications have become fixed throughout evolution, mostly in endemic regions where malaria is prevalent in the local population (Ko et al., 2011; Hodgson et al., 2014). Also, *P. falciparum* infection could trigger some host modifications related with epigenetic changes, thereby facilitating parasite evasion (Arama et al., 2018).

It is worth stressing that ligand-receptor interactions and environmental changes (i.e., temperature) influence parasite invasion of erythrocytes, as such interactions may trigger modifications in the parasite's signaling pathways that could induce changes in chromatin architecture and the formation and activation of transcription factor complexes governing gene transcription programmes (i.e., inducing sexual differentiation and gametocyte formation, or the expression of the genes required during invasion such as *eba175, eba140, eba181, rh4, rh2a*) (Stubbs et al., 2005; Rovira-Graells et al., 2012; Paul et al., 2015; Awandare et al., 2018). These transcriptional changes begin with nucleosome remodeling in regions containing the genes which must be expressed. Specific transcription factors then form complexes which recognize and bind to specific promoter motifs upstream of these genes (Balaji et al., 2005; Salcedo-Amaya et al., 2009). However, engaging transcription factors at the promoter is not sufficient to start gene transcription by itself, because these complexes also contain transcriptional repressors which must be modified post-translationally for transcription to proceed (Chêne et al., 2012). Such post-translational modifications are mediated by proteins expressed upon external stimuli (related to parasite internal signaling), enabling the parasite's adaptive changes, by transcription regulation, depending on a particular host's environment. On the other hand, it is worth highlighting that transcriptional regulatory changes might also be a consequence of stochastic events that could lead to selecting the fittest parasites; the real contribution of adaptive, stochastic or both mechanisms remains to be determined.

Studying such interactions and changes in the parasite-host relationship may facilitate a renewed approach to dealing with parasite infection; microarray, chromatin immunoprecipitation and sequencing technologies have accelerated most recent discoveries in this field, nevertheless, more studies are needed to characterize the complete set of factors affecting *P. falciparum*'s invasion of host cells. Further investigation is required regarding the signaling pathways activated by external stimuli which could play a role in the epigenetic and transcriptional changes affecting the expression of some invasion ligands related to invasion phenotypes. Such efforts will enable finding critical points which could be exploited for medical treatment and vaccines facilitating effective control of this disease.

## Author Contributions

MA-S conceived the work, drafted the manuscript and designed the figures. MAP and HC critically revised the manuscript for intellectual content. All the authors have read and approved the final manuscript.

### Conflict of Interest Statement

The authors declare that the research was conducted in the absence of any commercial or financial relationships that could be construed as a potential conflict of interest.

## Abbreviations

PV: Parasitophorous vacuole
ORF: open reading frame
WHO: World Health Organization
PIC: pre-initiation complex
AP2 or ApiAP2: apetala2 family of transcription factors
*Pf* AP2: *P. falciparum* apetala2 family of transcription factors
*Pf* AP2-I: *P. falciparum* apetala2 family - invasion
pfmsp5: *P. falciparum* merozoite surface protein 5 gene
Rh4: reticulocyte binding protein homolog 4
*Rh2b*: reticulocyte binding protein 2 homolog b
*Pf* BDP1: *P. falciparum* bromodomain 1
MSP1: merozoite surface protein 1
AMA-1: apical membrane antigen 1
*EBA175*: erythrocyte binding antigen-175
*EBA140*: erythrocyte binding antigen-140
*Pf* Alba: *P. falciparum* Alba-family protein
Alba: acetylation lowers binding affinity
Sir2: sirtuin 2
*Pf* RhopH3: *P. falciparum* high molecular weight rhoptry protein 3
*Pf* CDPK1*, P. falciparum* calcium-dependent protein kinase 1; Myb: myeloblatosis family of transcription factors
HMG: high-mobility-group
*Pf* Pk5: cdc2-related cyclin-dependent kinase
H3: histone 3
H2A: histone 2A
*Pf* TBP: *P. falciparum* TATA box binding protein
*Pf* TFIIE*, P. falciparum* transcriptional factor IIE; HMBP: high mobility group box proteins
HAT: histone acetyltransferase
HDAC: histone deacetylase
H3K9ac: histone 3 lysine 9 acetylated
GCN5: general control nonderepressible 5
*Pf* HP1: *P. falciparum* heterochromatin protein one
*EBA181*: erythrocyte binding antigen-181
mTOR: mammalian target of rapamycin
GalNAc: 3-α-N-acetylgalactosaminyltransferase
UDP: uridine diphosphate
Gal: 3-α-galactosyltransferase
DARC: Duffy antigen receptor for chemokines
*Pf* CnB: calcineurin B.
